# Reproducibility of QM/MM Calculations for the SARS-CoV‑2 Main Protease

**DOI:** 10.1021/acs.jctc.5c00841

**Published:** 2025-07-24

**Authors:** Xiaoli Sun, Ulf Ryde

**Affiliations:** a Department of Computational Chemistry, 5193 Lund University, Chemical Centre, PO. Box 124, Lund SE-221 00, Sweden; b Institute of Theoretical Chemistry, College of Chemistry, Jilin University, Changchun 130012, PR China

## Abstract

Combined quantum mechanics and molecular mechanics (QM/MM) calculations are a popular approach to study reaction mechanisms of enzymes. However, recently, the reproducibility of such calculations has been questioned, comparing the results of two software: NWChem and Q-Chem. Here, we continue and extend this study by including three additional software—ComQum, ORCA, and AMBER—using the same test case, the covalent attachment of the carmofur inhibitor to the catalytic Cys-145 residue of the SARS-CoV-2 main protease, using a quantum region of 83 atoms. We confirm that the various software programs give varying results for the reaction (Δ*E*) and activation (Δ*E* ^‡^) energies. The main reason for the variation is how charges around the cleaved bonds between the QM and MM regions are treated, i.e., the charge-redistribution scheme. However, there are still differences of ∼10 kJ/mol between different implementations of the same method in ComQum and ORCA. Some of these problems can be solved by calculating the final energies with larger QM systems. We show that energies calculated with the big-QM approach are reasonably converged if atoms within 8 Å of the minimal QM region are included (∼1400 atoms), solvent-exposed charged residues are neutralized, and the calculation is performed in a continuum solvent with a dielectric constant of 80. On the other hand, we show that different setups of the protein lead to even larger differences in the calculated energies, by up to 114 kJ/mol. Even if the same approach is used and the only difference is how water molecules are added (by random) to the crystal structure, energies differ by 18–57 kJ/mol. The results also strongly depend on how much of the surrounding protein and solvent are relaxed in the calculations. Therefore, it seems that for a solvent-exposed active site, QM/MM calculations with minimized structures cannot be recommended. Instead, methods that incorporate dynamic effects and calculate free energies seem preferable.

## Graphical abstract

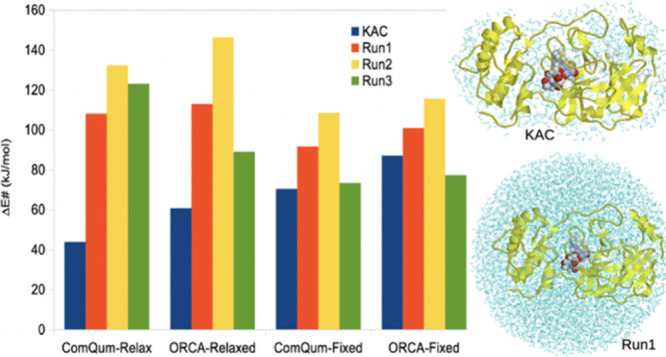

## Introduction

The combined quantum mechanical and molecular mechanical (QM/MM) method is a popular approach to study chemical reactions in solution and in biological macromolecules.^1,2^ It employs quantum mechanical (QM) calculations for a small portion of the system, where the chemical reaction takes place, called system 1 or the QM region in the following, whereas the remaining bulk of the system, called system 2 or the MM region, is treated by molecular mechanics (MM) calculations. Thereby, the method may combine the accuracy of the QM calculations with the speed of the MM calculations.

Many variants of QM/MM exist. In the additive scheme,^3^ the total energy is the sum of three terms:

$$E_{\mathrm{QM}/\mathrm{MM}}^{\mathrm{add}}=E_{\mathrm{QM}}^{1}+E_{\mathrm{MM}}^{2}+E_{\mathrm{QM}/\mathrm{MM}}^{1-2}$$ 1

where *E*_QM_^1^ is the QM energy of the QM region, *E*_MM_^2^ is the MM energy of the MM region and *E*_QM/MM_^1 – 2^ is the interaction energy between the two regions. In the subtractive scheme, the second and third terms on the right-hand side are different:

$$E_{\mathrm{QM}/\mathrm{MM}}^{\mathrm{sub}}=E_{\mathrm{QM}}^{1}+E_{\mathrm{MM}}^{12}-E_{\mathrm{MM}}^{1}$$ 2

*E*_MM_^12^ is the MM energy of the total system (i.e., both the QM and MM regions) and *E*_MM_^1^ is the MM energy of the QM region. The advantage of the additive scheme is that bonded MM parameters for the QM system are not needed, whereas the advantage of the subtractive scheme is that any QM and MM software can be employed without any modifications.

A second issue is how the electrostatic interactions between the QM and MM regions are treated (typically the most important interactions). In mechanical embedding (ME), they are treated by MM, employing MM charges for the QM system. This means that neither the QM region nor the MM region are polarized by each other. In electrostatic embedding (EE), a MM point-charge model of the MM system is included in the QM calculations, ensuring that the QM region is polarized by the MM region, but not vice versa. In polarized embedding (PE), both the QM and MM regions are polarized by each other. PE requires a polarizable MM force field and is normally obtained by including both MM point charges and polarizabilities in the QM calculations, solved self-consistently.^4^ Equations 1 and 2 above represent ME, but they can easily be modified for EE or PE by including point charges and possibly polarizabilities in the *E*_QM_^1^ term, and ensuring that the corresponding interactions are excluded from the other two terms on the right-hand side, to avoid double-counting. Currently, EE is the most popular approach, although the unbalanced polarization may lead to overpolarisation of the QM region.^5^

A third issue is how covalent bonds between the QM and MM regions are treated. The QM calculations require filled valences, so the QM region needs to be properly truncated. The most common approach is to terminate the QM region by a H atom (a hydrogen link atom, HL) for each cleaved bond, the hydrogen link-atom approach.^6^ Such calculations can be performed with any QM software, but it introduces several problems: The HL atoms are of an incorrect element, placed at incorrect positions and they give rise to spurious electrostatic interactions with nearby charges and polarizabilities in the EE and PE schemes, which may lead to inaccurate energies. Therefore, several approaches have been developed to remove or redistribute charges around the link atoms in the EE scheme.^5,7,8^ The subtractive scheme may correct some of the errors introduced by the HL atoms, but with limited success in practice.^3^ Other types of link atoms (halogens or special parametrized atoms) have been tested but are seldom used.^1^ The alternative is to use special localized orbitals at the cleaved bonds, e.g. the local self-consistent field or generalized hybrid orbital approaches.^1^ They require special QM codes and extensive parametrizations and are therefore used only in a few software.

Recently, Krylov and co-workers published a study with the title “How reproducible are QM/MM simulations?” (called KAC in the following).^9^ They showed that for the nucleophilic addition between the active-site Cys-145 residue in SARS-CoV-2 main protease (M^Pro^) and the covalent inhibitor carmofur, QM/MM implementations in the NWChem^10^ and Q-Chem^11^ software gave differences of up to 30 kJ/mol in the calculated QM/MM energies, even for single-point energies on the same coordinates with the same QM method and basis set. Naturally, it is very alarming if a computational method is not reproducible, although it is not completely unexpected considering the many issues involved in the implementation of QM/MM methods. However, it is of critical interest to understand why the results are so different and how these differences can be avoided.

Therefore, we here extend this study in several ways. First, we calculate QM/MM energies with three additional software (ComQum,^12,13^ ORCA^14^ and AMBER^15^) and try to explain the source of the differences. Second, we try to obtain converged energies for the reaction, which lead to an extension of our big-QM approach.^16^ Third, we examine how the results depend on different setups of the protein, involving both different philosophies and different random choices.

## Methods

### Protein Setup

Two sets of calculations were performed. In the first, we employed QM/MM structures obtained by KAC,^9^ viz. the 83REAC_density_espfit.pdb and 83PROD_density_espfit.pdb files included in their Supporting Information. They represent the reactant state (RS) and product state (PS) of the reaction. They involve 8151 atoms, including 306 amino-acid residues, the inhibitor and 1145 water molecules. They represent the protein with a single layer of water molecules surrounding most residues, but ∼ 10 Å of water molecules around the active site (cf. Figure 1a). The catalytic Cys-145 residue is negatively charged and the His-41 residue is positively charged and forms a hydrogen bond to SG in Cys-145 (2.37 Å in the RS structure; cf. Figure 2a). His-80 is protonated on the ND1 atom, whereas all the other His residues are protonated on NE2. The net charge is −3. Carmofur is solvent exposed and binds with the carbonyl carbon 3.44 Å from the sulfur atom of Cys-145.

**Figure 1:**
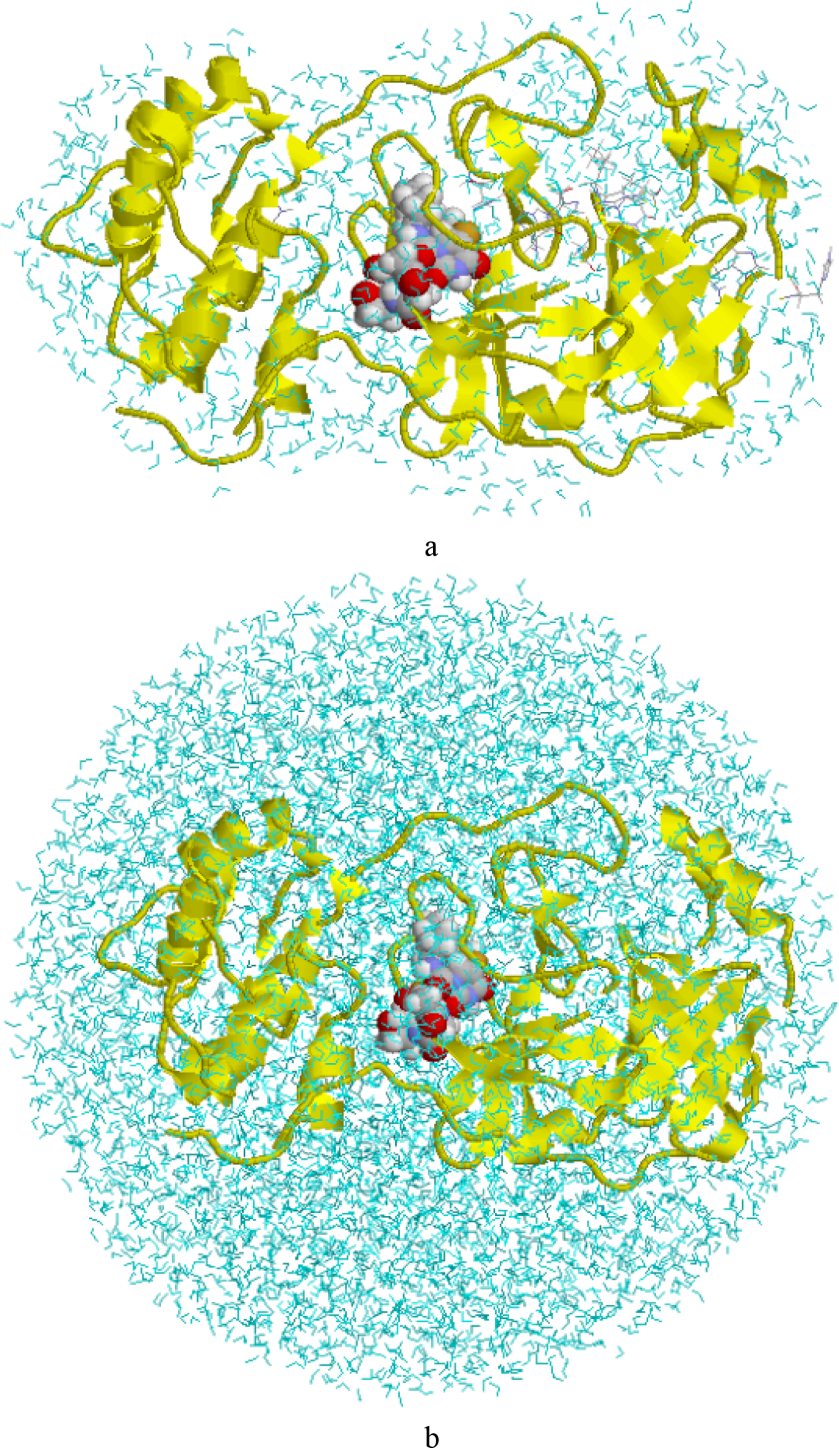
Setups used by (a) KAC and (b) us (Run1). The QM system is shown by space-filling spheres, the protein in yellow cartoon and water molecules in cyan wireframes.

**Figure 2:**
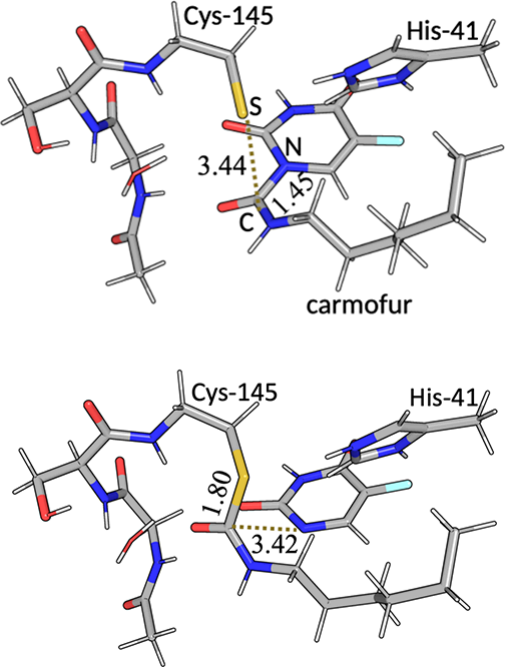
Structures of (a) RS and (b) PS, showing also the 83-atom QM region employed in the calculations. Distances are in Å. Carbon atoms are colored gray, oxygen red, nitrogen blue, hydrogen white, fluorine cyan and sulfur yellow.

To examine how the setup of the protein affects the results, we also made another setup of the protein, following our standard approach.^2^ We started from the same PDB file as KAC, 7BUY, showing M^pro^ in complex with carmofur (but in the PS state, after the formation of the S–C bond and dissociation of the fluoro-uracyl group) at 1.60 Å resolution.^17^ Five molecules of DMSO were deleted as well as two water molecules that partly overlap with carmofur. We added four water molecules from the structures of KAC, because they are in their larger QM system. A single additional water molecule in their QM system is close to a crystal-water molecule (HOH-571) and we used the crystal position for this molecule. All crystal-water molecules were kept. The structure was reverted to the RS state, i.e. the carmofur molecule was moved to the position in the RS file of KAC and the uracyl group was introduced. In this position, it overlaps somewhat with CG23 of Thr-25. Therefore, the side chain of this residue was allowed to move in the equilibration of the system.

The protonation states of all residues were determined from a detailed study of the hydrogen-bond pattern and the solvent accessibility. It was checked by the PROPKA^18^ and Maestro software.^19^ Following the suggestions of the latter software, we rotated the amide side chain of Gln-69, Asn-180 and Asn-277. All Arg, Lys, Asp, and Glu residues were assumed to be charged. Residues His-64 and 80 were protonated on ND1, His-41 was protonated on both the ND1 and NE2 atoms (following the suggestion by KAC; in the crystal structure, only protonation of NE2 is supported), whereas the remaining three His residues were modeled with a proton on the NE2 atom. There is no carboxy-terminal atom, so we kept this terminal neutral (in variance to the setup by KAC), whereas the amino-terminal Ser-1 residue was modeled with a –NH_3_^+^ group.

The protein was then solvated in a rectangular box of water molecules, extending at least 10 Å from the protein. Since the solvation of the protein is completely arbitrary, this procedure was repeated three times with different pre-equilibrated boxes,^20^ giving three different, but equivalent setups, called Run1, Run2 and Run3 in the following.

The solvated system was then subjected to 1000 step conjugated-gradient minimization, followed by 10 ps MD simulation without any bond-length constraints. Next, 1 ns MD with constant volume and 10 ns equilibration with constant pressure were run. Finally, a 10 ns simulated annealing calculation (up to 370 K) was run, followed by a minimization. In all the latter four simulations, bond lengths involving H atoms were constrained with the SHAKE algorithm,^21^ allowing a time step of 2 fs. In all simulations, only the added protons and water molecules (and the side chain of Thr-25) were allowed to move whereas the other atoms were kept at the crystal-structure positions with a harmonic constraint with a force constant of 41840 kJ/mol/Å^2^. Finally, the equilibrated structure was truncated to a spherical shape with a radius of 40 Å and the center of mass of the protein in the origin (cf. Figure 1b). After truncation, the three different setups involved 7126–7414 water molecules and 26094–26958 atoms in total. The net charge was – 4.

All MM calculations were performed with the Amber 22 software.^15^ For the protein, we used the AMBER ff19SB force field^22^ and water molecules were described by the TIP3P model.^23^ Carmofur was described by the GAFF 2.1 ^24^ force field using AM1-BCC charges^25^ calculated with antechamber.^15^ Atom types and charges of carmofur are given in Table S1 in the Supporting Information.

### QM/MM Calculations

We used three software for QM/MM calculations, ComQum,^12,13^ ORCA 5.0.4 ^14^ and AMBER.^15^ All three software use the HL approach: When there is a bond between systems 1 and 2 (a junction), the QM system is capped with hydrogen atoms (HL), the positions of which are linearly related to the corresponding carbon atoms (carbon link atoms, CL) in the full system.^6,12^ However, the detailed implementation may differ between the three software. The one used by ComQum is described in the Supporting Information.

The QM system was the same in all calculations and was taken from KAC. It involved carmofur, His-41 (modeled as methylimidazole), all atoms in Cys-145 (except the backbone O atom), Ser-144 and Gly-143 and a terminating backbone CH_3_CO– group from Asn-142, as well as a water molecule that donates a hydrogen bond to SG of Cys-145 and receives a hydrogen bond from Ser-144. It consisted of 83 atoms in total and is shown in Figure 2. The QM method was also the same as in KAC, PBE0 ^26^ and the 6–31G* basis set.^27^ DFT-D3 dispersion corrections were included with zero damping.^28^

ComQum combines the Turbomole and Amber software. By default, it employs a subtractive scheme with electrostatic embedding and van der Waals link-atom corrections.^3^ The total QM/MM energy in ComQum is calculated as^12,13^

$$E_{\mathrm{QM}/\mathrm{MM}}=E_{\mathrm{QM}1+\mathrm{ptch}2}^{\mathrm{HL}}+E_{\mathrm{MM}12,q_{1}=0}^{\mathrm{CL}}-E_{\mathrm{MM}1,q_{1}=0}^{\mathrm{HL}}$$ 3

where *E*_QM1+ptch2_^HL^ is the QM energy of the QM system truncated by HL atoms and embedded in the set of point charges modeling system 2 (but excluding the self-energy of the point charges). *E*_MM1,q_1_=0_^HL^ is the MM energy of the QM system, still truncated by HL atoms, but without any electrostatic interactions. Finally, *E*_MM12,q_1_=0_^CL^ is the classical energy of all atoms (in both the QM and MM regions) with CL atoms and with the charges of the QM region set to zero (to avoid double-counting of the electrostatic interactions). No cutoff is used for any of the QM/MM energy terms. Optionally, all MM atoms within 6 Å of the QM region (741–891 atoms in total, viz. Thr25–Asn28, Cys38–Cys44, Met49, Pro52, Ty54, Cys85, Ala116–Gly120, Tyr126, Ser139–Asn142, Cym145–Ser147, Met162–Glu166, His172, Phe181, Val186–Gln189, Gln192, as well as 50 (Run1), 59 (Run2) or 57 (Run3) water molecules; cf. Table S2) were allowed to relax by 1000 steps of conjugate-gradient minimization in each step of the QM/MM geometry optimization, keeping the QM system fixed. The QM calculations were performed with the Turbomole 7.7 software.^29^

With ORCA, we employed the default additive scheme with EE. Optionally 534–616 atoms in the surrounding MM system were optimized (cf. Table S2). The MM system was described by converting the AMBER parameter and topology file (used by ComQum) to ORCA format using the orca_mm -convff -AMBER prmtop command. A sample input file is shown in Table S3.

QM/MM calculations with AMBER were employed only for single-point energy calculations using the input coordinates from KAC. The QM/MM calculations involve an additive scheme and EE. They employ external QM calculations using the ORCA software. A sample input file is given in Table S4.

### Charge-Redistribution Schemes

As mentioned above, EE-QM/MM approaches may differ in which MM atoms are included as point charges in the QM calculations. We have tested several of these. To explain the variation, we use the following nomenclature (cf. Figure 3): We assume that there is a bond between an atom Q1 in the QM system and M1 in the MM system. M1 is replaced by a HL atom in the QM calculations and no other atoms in the MM system are included in the QM calculation (as real QM atoms). M1 is the atom called CL above. Atoms covalently bound to Q1 in the QM system are called Q2. Those covalently bound to Q2 are called Q3 (excluding the Q1 atoms). Likewise, MM atoms covalently bound to M1 are called M2, and those covalently bound M2 (excluding M1) are called M3.

**Figure 3:**
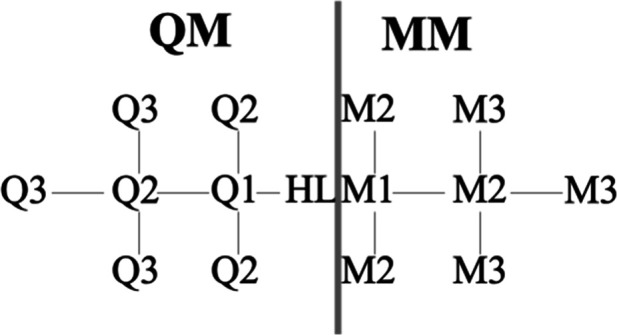
Nomenclature of atoms around covalent bonds spanning the QM and MM systems in the hydrogen link-atom approach. M1 is the same as the CL atom.

With this nomenclature, the various tested charge-redistribution schemes are defined in the following way:^5^

- Z0: All MM atoms are included in the point-charge model, including the M1 atoms.
- Z1: The charges of the M1 atoms are excluded.
- Z2: The charges of the M1 and M2 atoms are excluded.
- Z3: The charges of the M1, M2, and M3 atoms are excluded.
- Zz2: The charges of the M1 and M2 atoms are excluded. The sum of the charges on the remaining MM in the junction residue is set to zero by adding the same increment to all charges.
- RCD (the redistributed charge and dipole method^7^): Charges of the M1 atoms are redistributed over all the M2 atoms, keeping the bond dipole constant by adding a compensating charge at the bond midpoint between each M1 and M2 atom.
- CS (the charge-shift scheme^8^): The M1 charge is redistributed, keeping the bond dipole by compensating charges on the M2 atoms and two point charges placed on each side of M2 along the M1–M2 bond. The detailed implementation in ComQum is described in ref 5.

For Z1–Z3, we also tested to redistribute the sum of the deleted charges evenly on the other MM atoms in that residue. These approaches are called DZ1, DZ2, and DZ3.

The RCD and CS schemes were developed for force fields with charge groups (i.e., where the charges of e.g. CH_3_ and CH_2_ sum up to zero; used e.g. in CHARMM). In the AMBER ff19SB force field^22^ used in the current calculations, this is not the case (only charges of the entire residue sum up to an integer). In ComQum, M1 (= CL) is considered to be the same atom as Q1 (HL), but with a different element and position in the QM or MM calculations, respectively. Then, it seems illogical to include the M1 charge in the point-charge model or to redistribute it onto the other charges. Therefore, Z1 is the default charge-redistribution scheme in ComQum,^5^ whereas CS is default in ORCA.

### Big-QM Calculations

Previous studies have shown that QM/MM energies strongly depend on the size of the studied QM system.^5,16^ Therefore, we have developed the big-QM approach to ensure that QM/MM energies are converged.^30,31^ This approach involves single-point QM/MM calculations on optimized QM/MM structures with a very large QM region, including all atoms within a certain distance *r* of a minimal QM region and all buried charged-residues (no such residues are present in M^Pro^), and moving junctions at least two residues away from the original QM system. Previous studies have used *r* values of 4.5 or 6 Å.^30,31^ Here, we used *r* = 6, 8, and 10 Å (from the 83-atom QM region) to judge the convergence of the big-QM energies. The QM calculations were still performed at the PBE0-D3/6–31G* level of theory, but they utilized the multipole-accelerated resolution-of-identity J approach (marij keyword in Turbomole).^32^

Two approaches were used to correct the big-QM energies for interactions with the surroundings. In the first, we used a point-charge model of the surroundings and added a MM correction:

$$E_{\mathrm{bigQM}}^{\mathrm{EE}}=E_{\mathrm{bigQM}+\mathrm{ptch}2}^{\mathrm{HL}}+E_{\mathrm{MM}12,q_{\mathrm{bigQM}}=0}^{\mathrm{CL}}-E_{{\mathrm{MM}}_{\mathrm{bigQM}},q_{\mathrm{bigQM}}=0}^{\mathrm{HL}}$$ 4

i.e. a standard EE QM/MM energy (cf. eq 3) but with the big-QM system as the QM region. In the second, we instead performed the big-QM calculations without the point-charge model in a COSMO continuum solvent as implemented in Turbomole.^33^ Default optimized COSMO atomic radii were applied,^34^ with a water solvent radius set to 1.3 Å for the construction of the solvent-accessible surface cavity. The calculations employed a dielectric constant (ε) of either 4 or 80.

## Result and Discussion

As discussed in the Introduction, KAC reported extensive variations between QM/MM results obtained with different QM/MM software.^9^ Here, we extend this study to three other software and try to understand and explain these differences. This is done in several steps, described in the following subsections. First, we perform single-point calculations on structures from KAC and try to obtain a reference results, using an updated version of the big-QM approach. Next, we study optimized structures and examine how the results depend on the setup of the protein.

SARS-CoV-2 main protease (M^pro^), also called the 3-chymotrypsin-like protease, cleaves the two polyproteins of SARS-CoV-2 at 11 sites.^35^ It is a cysteine protease, employing Cys-145 as a nucleophile, activated by His-41. It is a promising drug target because it shares no homology with any human proteases.^36^ Numerous studies have addressed covalent inhibitors of this enzyme.^37^ Several computational studies of the binding of covalent inhibitors to M^pro^ have been published both before^38^ and after^39^ KAC.

Following KAC, we study a one-step nucleophilic reaction of the SG atom of Cys-145 with the carbonyl carbon atom of the inhibitor carmofur, forming a covalent intermediate and a leaving fluoro-uracil group (Figure 2). Thus, the reactant state (RS) involves carmofur, a deprotonated Cys-145 and a doubly protonated His-45 (other studies have indicated that this is probably not the resting state of the enzyme; instead an initial endergonic proton transfer is needed;^39^ however, this does not matter in this study where different QM/MM approaches are compared). The product state (PS) contains the covalent adduct and a negatively charged fluoro-uracil group. We also study the transition state (TS) for this reaction.

### Single-Point Calculations

To start with, we concentrate on two structures optimized by KAC,^9^ viz. the structures of the RS and PS, obtained with a 83-atom QM system and optimized with NWChem, shown in Figure 2. In the RS, SG of Cys-145 is 3.44 Å from the carbonyl C on carmofur (simply called S–C in the following) and the bond between this C atom and its neighbor N atom in the fluoro-uracil ring is 1.45 Å (called C–N in the following). In the PS, there is a bond between SG and C, 1.80 Å, and the fluoro-uracil ring has started to dissociate with a C–N distance of 3.42 Å. These structures gave a QM/MM energy difference of 30 kJ/mol when calculated with NWChem or Q-Chem.^9^ We used these two structures and calculated single-point energies with three different software: ComQum,^12,13^ ORCA^14^ and Amber.^15^ We used the same QM method and basis set (PBE0-D3/6–31G*), and also the same MM charges (from AMBER ff99) as in KAC.

With default setting, we get reaction energies (Δ*E*) of –114, –96 and –115 kJ/mol with ComQum, ORCA and Amber, respectively (cf. Table 1). This slightly extends the energies observed with NWChem and Q-Chem, –78 and –108 kJ/mol, increasing the range to 37 kJ/mol.

**Table 1:** Δ*E* (kJ/mol) and Energy Components for the Various Variants of QM/MM in Five Different Software for Single-Point Calculations on the KAC Structures^a^

		Δ*E* (kJ/mol)			
software	variant	*E*_QM/MM_	*E*_QM1_	*E*_MM_	*E*_QM_
Q-Chem^9^		–108	–71	–37	–22
NWChem^9^		–78	–41	–37	–23
Amber		–115	–72	–42	
ComQum	default = Z1	–114	–74	–41	–21
	CS	–87	–46	–41	
	RCD	–82	–41	–41	
	Z0	–62	–21	–41	
	Z1	–114	–74	–41	
	Z2	–103	–62	–41	
	Z3	–90	–49	–41	
	DZ1	–107	–66	–41	
	DZ2	–90	–49	–41	
	DZ3	–84	–44	–41	
	ZZ2	–97	–56	–41	
	Add	–115	–74	–41	
	MEA	–48	–21	–27	
	MEE	–158	–21	–137	
Orca	default = CS	–96	–62	–34	–21
	DLAD	–95	–62	–33	
	DLB	–97	–62	–35	
	CS	–96	–62	–34	
	RCD	–74	–40	–34	
	Z0	–72	–38	–34	
	Z1	–109	–75	–34	
	Z2	–95	–61	–34	
	Z3	–82	–49	–34	
	MEA	–80	–21	–58	
	MEE	–119	–21	–98	

a *E*_QM/MM_ is the total QM/MM energy. *E*_QM1_ and *E*_QM_ are the QM energies of the QM region with or without the point-charge model (if any), respectively. *E*_MM_ is ***E***_MM_^**12**^ – *E*_MM_^**1**^for the subtractive calculations (cf. eq 2; ***E***_MM_^**12**^ is the MM energy of the total system and ***E***_MM_^**1**^is the MM energy of the QM region) and ***E***_MM_^**2**^ + *E*_QM/MM_^**1–2**^for the additive calculations (cf. eq 1; ***E***_MM_^**2**^is the MM energy of the MM region and ***E***_QM/MM_^**1–2**^is the interaction energy between the two regions). CS, RCD, Z0–Z3, DZ1–DZ2 and ZZ2 are different charge-redistribution schemes, explained in the Methods section. Add is additive QM/MM in ComQum. MEA and MEE are mechanical-embedding QM/MM with either standard Amber charges or ESP charges from the QM calculation. DLB indicates that the Q1–M1 bonds and DLAD indicates that the Q2–Q1–M1 angles and Q3–Q2–Q1–M1 torsions are included in the *E*_MM_ term.

With ComQum, we can also test several variants of QM/MM. The default implementation is subtractive QM/MM with EE, van der Waals HL corrections and the Z1 charge-redistribution scheme. The corresponding additive implementation gives nearly the same results (−115 kJ/mol; the difference is only 0.1 kJ/mol), as has also been observed before.^3^ The difference is caused by a change from using HL to CL atoms in the *E*_MM1,q_1_=0_^HL^ term in eq 3.

On the other hand, shifting to ME has a very large effect on Δ*E* and it also depends strongly on what charges are used for the QM atoms. Using standard AMBER charges for the protein (and the original AM1-BCC charges for carmofur; called MEA in Table 1) gives a much less negative Δ*E*, –48 kJ/mol, whereas using ESP charges from a single-point DFT calculation on the structures (MEE), gives a much more negative Δ*E*, –158 kJ/mol.

Next, we tested ten different charge-redistribution schemes (i.e., different ways to treat the charges around the HL atoms; explained in the Methods section). They gave results varying between –82 and –114 kJ/mol, with the default Z1 method giving the most negative results and the RCD method giving the least negative result. In fact, the Z0 method gives an even less negative result, –62 kJ/mol, but this is an inconsistent method in our eyes, because it includes the charge of the CL atom, which is the same as the HL atom, i.e. an atom interacts with itself. Still, together, these results show that different charge-redistribution schemes can give results that span the full range of results observed by the various software.

Naturally, all these charge-redistribution schemes can also be used with the additive QM/MM method in ComQum. However, as mentioned above, the subtractive and additive schemes give results that agree within 0.1 kJ/mol, so we do not include those results in Table 1.

Likewise, we tested nine different QM/MM variants in ORCA. The default implementation is additive QM/MM with EE and the CS charge-redistribution scheme. There are two additional variants that differ in whether the Q1–M1 bonds (DLB; cf. Figure 3), Q2–Q1–M1 angles and Q3–Q2–Q1–M1 torsions are included in the *E*_MM_ term (DLAD; in additive ComQum, none of those terms are included). Fortunately, it can be seen that these terms have only a minimal effect on Δ*E*, ± 1 kJ/mol.

Mechanical embedding has a significant effect on Δ*E*, but smaller than for ComQum, –80 kJ/mol. It is unclear from the manual what charges are used for the QM region. However, providing the ESP charges from the ComQum calculation with ME changes the results to −119 kJ/mol, indicating that the calculations employ the charges provided in the prmtop file.

We also tested the six different charge-redistribution schemes implemented in ORCA. They give Δ*E* energies varying from –72 to –109 kJ/mol. As with ComQum, Z1 gives the most negative Δ*E*, and Z0 and RCD give the least negative energies. With the same charge-redistribution schemes, the Δ*E* results of ORCA and ComQum agree within 10 kJ/mol (Figure 4). However, for CS and Z0, ORCA gives 9–10 kJ/mol more negative results, whereas for the other four approaches, ORCA instead gives 6–8 kJ/mol less negative results. As an effect, the range of the ORCA results (−72 to –109 kJ/mol) is smaller than that of ComQum (−62 to −114 kJ/mol for the same methods). This probably reflects details in the implementation of the additive approach (and possibly also in the charge-redistribution schemes) and the treatment of the HL atoms.

**Figure 4:**
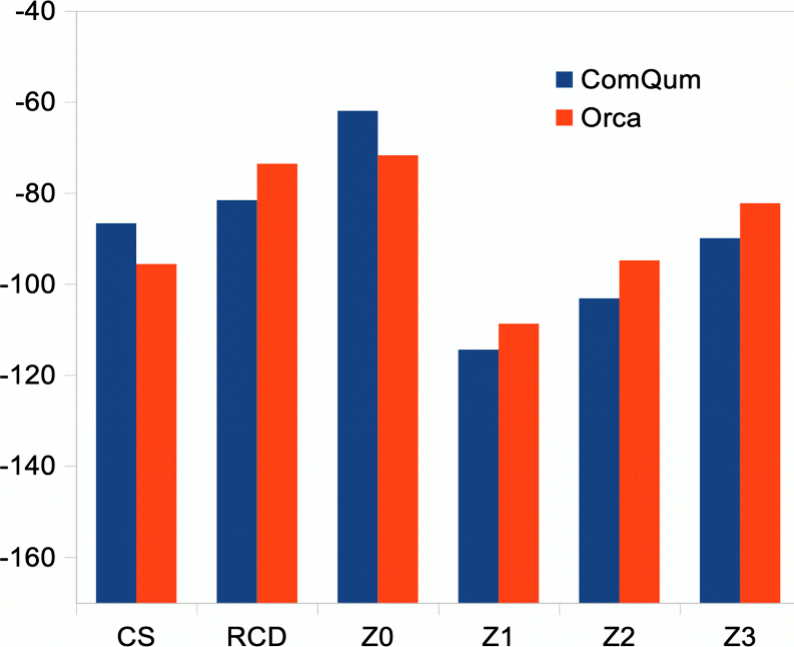
Comparison of QM/MM Δ*E* energies (kJ/mol) for similar charge-redistribution-schemes of ComQum and ORCA for the single-point calculations on the KAC structures.

Some further understanding of the problems can be obtained by studying the various QM/MM components (also in Table 1). QM calculations in vacuum (i.e., without the point-charge model, Δ*E*_QM_ in Table 1) give Δ*E* = –21 kJ/mol for both ComQum and ORCA (they differ by 0.2 kJ/mol). This is 1–2 kJ/mol less negative than what KAC reported for NWChem (−23 kJ/mol) and Q-Chem (−22 kJ/mol) and shows that the QM software give results in reasonable agreement.

The differences are larger for the QM calculation with the MM point charges (Δ*E*_QM1_ in Table 1), which is –71 to –74 kJ/mol for Q-Chem, AMBER and ComQum, but –62 kJ/mol for ORCA and –41 kJ/mol for NWChem. With the same charge-redistribution scheme, ComQum and ORCA agree within 2 kJ/mol for Z1–Z3 and RCD, but differ by 16–17 kJ/mol for CS and Z0. Apparently, the latter two approaches are implemented differently in the two software.

The MM contribution to Δ*E* (Δ*E*_MM_ in Table 1) is –41 to –42 for ComQum and AMBER, –37 kJ/mol for Q-Chem and NWChem, and –34 kJ/mol for ORCA. The latter (of course) does not change for the various charge-redistribution schemes, but differs between the various ME variants (Δ*E*_QM1_ differs between EE and ME, but is the same for the ME variants). It should be noted that it is quite unusual that the MM contribution to Δ*E* is so large for EE-QM/MM, half to one-third of the total energy. This indicates that the QM region is too small or that the MM region is not enough relaxed.

Finally, we note that if the QM region is optimized in vacuum, Δ*E* becomes much smaller, –3 kJ/mol. Moreover, the reactant state is unstable – if optimized freely, the proton on His-41 goes to the deprotonated Cys-146, forming a neutral reactive dyad (that state is 49 kJ/mol more stable than the zwitterionic state).

In conclusion, it is clear that details in the QM/MM implementation affects the energies, in particular the embedding approach and the charge-redistribution scheme. Consequently, it is of interest to know which result is most correct. This is the aim of next section.

### Single-Point Results with Larger QM System

The large variation of the Δ*E* result as a function of the charge-redistribution scheme indicates that the broken bonds are too close to the reactive center. Thus, a larger QM system is needed to obtain more reliable results. In principle, a single-point QM calculation of the entire MM model with 8151 atoms would give the correct QM result for these structures. But such extensive QM calculations are currently not possible. Moreover, it can be discussed how relevant such a result is for the real reaction in the protein. KAC’s model contains essentially only a single shell of solvent molecules around the protein. This will most likely overestimate the effect of solvent-exposed charges.

We have suggested the big-QM approach to obtain QM/MM energies that are converged with respect to the size of the QM region.^30^ According to this procedure, all atoms within a certain distance *r* from a minimal QM region (which in our case is the standard 83-atom QM region) are included in the QM calculations, in addition to all buried charged groups (none in M^pro^) and the backbone of three residues on both sides of the standard QM region (i.e., residues 38–44 and 139–148). To study the convergence of the big-QM calculations, we employed three different values of *r*, 6, 8, and 10 Å. We also tested to obtain the QM region with the distance criterion based on either RS or PS (since the coordinates are different, they give slightly different results). The QM regions involve 856, ∼1400 and ∼1875 atoms (cf. Figure S1 in the Supporting Information). Moreover, we tested to either include the remaining MM atoms with EE and a standard QM/MM MM term (eq 4) or by employing a COSMO continuum-solvation model with a dielectric constant of 4 or 80 (and no point-charge model).

The results of these Δ*E* calculations are collected in Table 2. It can be seen that the results are not fully satisfying. With all three treatments of the surroundings (EE, ε = 4 and ε = 80 in Table 2), Δ*E* keeps on increasing with *r*, from –69 to –127, –86 to –102 or –95 to –120 kJ/mol for the three approaches, i.e. ranges of 59, 16, and 26 kJ/mol. There are also rather large differences between big-QM regions selected based on the RS or PS structures, by 10–15 kJ/mol for EE, but it decreases to 0–4 kJ/mol for ε = 80.

**Table 2:** Big-QM Δ*E* Energies and Energy Components (all in kJ/mol) for the Single-Point Calculations on the KAC Structures Using Three Different Radii (*r =* 6, 8, and 10 Å)^a^

	Δ*E*_BigQM_						
	EE	ε = 4	ε = 80	ε = 80/neu	Δ*E*_QM_	Δ*E*_QM1_	Δ*E*_MM_
BigQM-6	–69	–86	–95	–95	–72	–73	–4
BigQM-8	–102	–101	–110	–108	–85	–92	9
BigQM-8-PS	–87	–95	–106	–102	–77	–97	–10
BigQM-10	–127	–102	–120	–114	–102	–102	26
BigQM-10-PS	–137	–116	–120	–114	–108	–142	–5

a The four big-QM variants treat the surroundings with an EE-QM/MM model (eq 4; EE) or with a COSMO continuum-solvent model with a dielectric constant of either 4 or 80 (ε = 4 or ε = 80). In the ε = 80/neu calculation, solvent-accessible charges are neutralized. The big-QM regions were selected based on the RS structures, except for the BigQM-8-PS and BigQM-10-PS calculations, for which the PS structure was instead used. Δ*E*_QM_ and Δ*E*_QM1_ are QM energies for the big-QM system, calculated either without or with a point-charge model of the surroundings (i.e. the latter is *E*_bigQM + ptch2_^HL^ in eq 4). Δ*E*_MM_ = *E*_MM12,q_big–QM_=0_^CL^ – *E*_MM_big–QM_,q_big–QM_=0_^HL^ from eq 4. The Z1 charge redistribution scheme was used for the EE calculations.

A detailed investigation of the calculations indicate that the problem comes primarily from four solvent-exposed charged residues, Arg-188, Asp-48, Glu-166 (all three not present when *r* = 6 Å) and Lys-61 (included only when *r* = 10 Å; Arg-40 and Asp-187 are present in all QM systems, but they form an ionic pair). Such problems have been observed before.^40^ They have been solved by scaling down or removing the charges of these residues or by neutralizing them. We decided to test the latter approach, which is less drastic. Thus, each residue was neutralized by either deleting the HH22 (Arg-188) or HZ3 (Lys-61) atoms or by protonating the OD1 (Asp-48) and OE2 atoms (Glu-166; the atoms were chosen as those that disturb the hydrogen-bond structure the least). This gave slightly damped results, –95 to –114 kJ/mol, with a range of 19 kJ/mol (column ε = 80/neu in Table 2). In particular, the results of *r* = 8 and 10 Å differ by only 6 kJ/mol and there is no difference between big-QM regions selected based on the RS or PS structures for *r* = 10. Thus, we consider –114 kJ/mol as our best Δ*E* result for the KAC structures, in good agreement with the QM/MM results obtained with Q-Chem, Amber, ComQum with Z1, DZ1 and the additive scheme, as well as ORCA with Z1 and MEE, but this may very well be fortuitous for this single test system.

We also tested the ten different charge-redistribution schemes for the EE big-QM calculations (with COSMO, there is no point-charge model of the surroundings; Z1 was used for the calculations in Table 2). The results in Figure 5 show that for the smaller *r* = 6 Å, the variation in the big-QM energies for the various charge-redistribution schemes is actually larger than for the QM/MM calculations (−72 to –167 kJ/mol), probably reflecting that the number of HL atoms is much larger (41 compared to 4) and some of them are still too close to the reactive atoms. Z2 gives the most negative and deviating Δ*E*. For larger radii, the variation is reduced, e.g. to –85 to –131 kJ/mol *r* = 8 and 10 Å. For these big-QM systems, the number of HL atoms is almost the same, 42 for both, but they move successively away from the reactive center as the big-QM system grows. It can be seen that the Z0, Z2 and DZ2 schemes show the largest variation with different radii, whereas ZZ2, Z3, CS and RCD show the smallest variation. Although the variation is quite large, there is a clear tendency that the results converge toward what was obtained with big-QM and COSMO. For example, the average of the Δ*E* results with the ten charge-redistribution schemes is –97, –121 and –108 kJ/mol, for *r* = 6, 8, and 10 Å, respectively, compared to –95, –108 and –114 kJ/mol for big-QM with ε = 80 and neutralized systems. Thus, the big-QM methods are reasonably consistent and the results with the COSMO continuum-solvent are clearly more stable.

**Figure 5:**
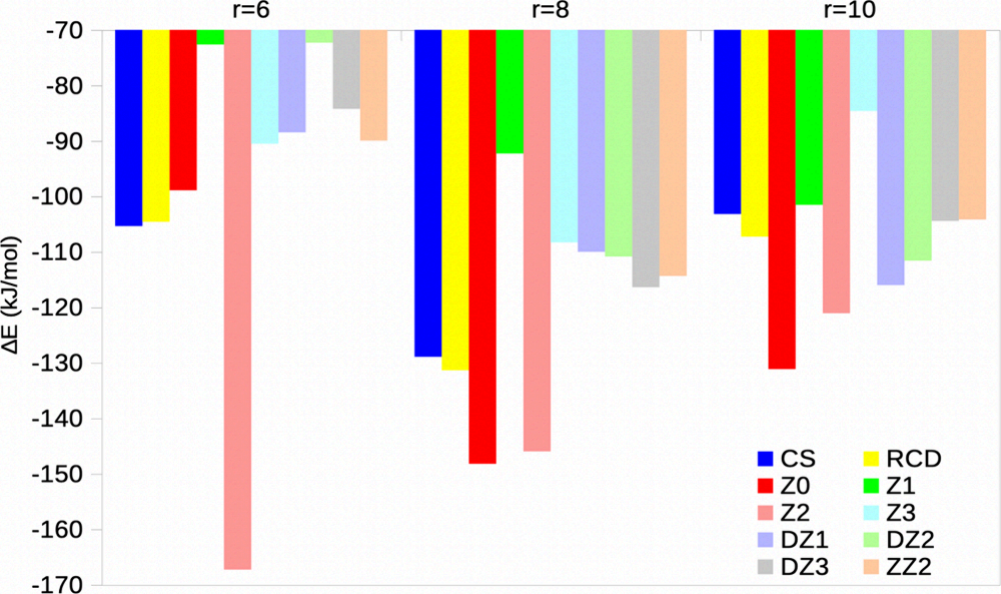
Dependence of the big-QM EE Δ*E* reaction energy on ten difference charge-redistribution schemes for the single-point calculations on the KAC structure and three different values of *r* (6, 8, or 10 Å).

### Optimized Structures

Next, we performed QM/MM optimizations of the nucleophilic reaction with ComQum and ORCA using default settings. We employed four different setups. First, we used the setup from KAC, involving the protein and a single shell of water molecules (8151 atoms; Figure 1a). Second, we tried the setup normally used in our group, i.e. the full protein in a sphere of water molecules (Figure 1b). To estimate how much the results depend on the setup, we performed three equilibrations of the protein, using three different (random) solvent boxes (26094–26958 atoms), but keeping the protein essentially fixed at the starting crystal structure in all. The four setups are called KAC, Run1, Run2 and Run3 in the following. For ComQum, the transition state (TS) was found by a relaxed scan. For the KAC system, it was enough to follow the S–C distance between the SG atom of Cys-145 and the amide carbon atom in carmofur. However, for the other systems, this did not lead to any release of the fluoro-uracil product. Therefore, we instead used the anharmonic combination of the S–C and the C–N distances. With ORCA, TS was obtained with the nudged elastic band (NEB) approach,^41^ but for KAC we also tried a scan of the S–C distance and a full TS-search, showing that the three approaches give closely similar energies. With both software, the reactions were studied either by keeping the surroundings fixed to the starting crystal structure or by relaxing 500–800 MM atoms.

KAC reports activation energies (Δ*E* ^‡^) of 36 (NWChem) or 23 kJ/mol (Q-Chem single-point calculation on the NWChem structure). When we optimized the KAC structure with ComQum and relaxed surroundings, we get a Δ*E* ^‡^ of 44 kJ/mol (Table 3), which is similar to the NWChem result. The Δ*E* results also coincide, –78 kJ/mol, indicating that the two software use similar approaches and charge-redistribution schemes. However, there are rather large differences in the TS structure (also Table 3): The S–C bond length is 2.40 Å and the N–C bond length is 1.50 Å, compared to 1.95 and 1.74 Å in KAC. This can be caused by the starting position of carmofur in the two structures. In RS, the ComQum S–C distance is 3.73 Å, compared to 3.44 Å in KAC (the N–C bond length is 1.45 Å with both software). In PS, the C–S distance is 3.57 Å with ComQum and 3.42 Å in KAC, whereas the S–C bond length is 1.79 Å.

**Table 3:** Results (Δ*E* ^‡^ and Δ*E* in kJ/mol; S–C and C–N Distances in Å for the RS, TS and PS Structures) for the QM/MM Geometry Optimizations Using Four Different Setups (KAC or Run1–3), with Either Fixed or Relaxed MM Surroundings (Relax) and Using Three Different Software (NWChem, ComQum or ORCA)

			Δ*E* ^‡^	Δ*E*	RS		TS		PS	
setup	relax	software	kJ/mol	kJ/mol	S–C	C–N	S–C	C–N	S–C	C–N
KAC	yes	NWChem	36	–78	3.44	1.45	1.95	1.74	1.79	3.42
		ComQum	44	–78	3.73	1.45	2.40	1.50	1.79	3.57
		ORCA	61	–57	3.43	1.45	2.00	1.65	1.80	3.37
	no	ComQum	70	–16	3.39	1.44	1.95	1.75	1.81	3.25
		1 fix^a^	70				1.95	1.65		
		ORCA	85	6	3.44	1.45	2.02	1.63	1.81	3.23
		TS^b^	87				1.97	1.75		
		scan^c^	87				1.94	1.68		
Run1	yes	ComQum	108	–1	5.36	1.45	1.95	1.85	1.78	3.55
		ORCA	113	57	3.71	1.45	2.85	1.46	1.82	2.92
	no	ComQum	92	–2	3.39	1.44	1.91	1.86	1.79	3.09
		ORCA	101	80	3.42	1.45	1.94	1.79	1.81	2.77
Run2	yes	ComQum	132	–19	3.74	1.44	2.03	1.73	1.79	3.50
		ORCA	146	25	3.64	1.45	2.22	1.97	1.80	3.23
	no	ComQum	108	18	3.50	1.45	1.95	1.85	1.79	3.15
		ORCA	115	37	3.54	1.45	1.91	1.90	1.80	3.15
Run3	yes	ComQum	123	–17	3.53	1.44	2.01	1.70	1.78	3.42
		ORCA	89	25	3.46	1.45	1.93	1.75	1.80	3.04
	no	ComQum	73	0	3.43	1.45	1.88	1.77	1.79	3.03
		ORCA	77	47	3.50	1.45	2.21	1.55	1.79	3.22

a TS obtained with only the S–C bond length fixed in ComQum, rather than the anharmonic combination of the S–C and C–N bond lengths.

b TS obtained with a full TS optimization with ORCA, rather than a nudged elastic band (NEB) calculation.

c TS obtained from a scan of the S–C bond length in ORCA.

On the other hand, ORCA gives a higher Δ*E* ^‡^ of 61 kJ/mol and a less negative Δ*E* of –57 kJ/mol. If the surroundings are not allowed to relax, the energies become significantly more positive: With ComQum, Δ*E* ^‡^ is 70 kJ/mol and Δ*E* is –16 kJ/mol, both with a single S–C bond restraint and with the anharmonic combination of S–C and C–N. With ORCA, the corresponding energies are 85 and +6 kJ/mol. A single bond-length restraint gives the same Δ*E* ^‡^ as a full TS geometry optimization (87 kJ/mol), whereas a NEB calculation gives a 2 kJ/mol smaller barrier. However, the S–C distances vary more, 1.97, 1.94, and 2.02 Å, as does the C–N distance, 1.75, 1.68, and 1.63 Å, showing that the potential surface is quite flat around the TS (1.95 and 1.75 Å with ComQum).

Interestingly, the results obtained with our setup are quite different. For Run1, the ComQum results with relaxed surroundings are 108 and –1 kJ/mol, i.e. 64 and 77 kJ/mol more positive than those obtained with the KAC setup, respectively. Δ*E* ^‡^ with ORCA is quite similar, 113 kJ/mol, but Δ*E* is even more positive, 57 kJ/mol. The corresponding results with fixed surrounding are more similar to those with the relaxed surroundings than for the KAC setup, 92 and –2 kJ/mol with ComQum and 101 and 80 kJ/mol with Orca.

Discouragingly, the other two random solvation setups (Run2 and Run3) give quite different results (cf. Table 3). The energies obtained with the three setups (Run1–Run3) show a range of 24–57 kJ/mol for Δ*E* ^‡^ (e.g., 108–132 kJ/mol for ComQum with relaxed surroundings) and 18–42 kJ/mol for Δ*E* (e.g., –1 to –19 kJ/mol for ComQum with relaxed surroundings). The range is always larger with ORCA (average 42 kJ/mol) than with ComQum (24 kJ/mol), indicating that Z1 charge-redistribution scheme or the subtractive scheme gives more stable results than the CS charge-redistribution scheme or the additive scheme.

Table 3 also shows how the geometry of the RS, TS and PS vary in the 16 different QM/MM optimizations. It can be seen that the two covalent bonds (C–N in RS and S–C in PS) show only minimal variation (0.02 and 0.04 Å among the four setups, respectively). RS shows one prominent outlier (Run1 with ComQum and relaxed surroundings), for which the S–C distance is more than 1.6 Å longer than for the other calculations (which show a variation of 3.39–3.74 Å). PS shows a more even, but larger, distribution in the C–N distance, 2.77–3.57 Å.

For the TS, four calculations differ from the others (Run1 with ORCA and relaxed surroundings, KEA with ComQum and relaxed surroundings, Run2 with ORCA and relaxed surroundings, and Run3 with ORCA and fixed surroundings). The other calculations (including NWChem) give S–C distances of 1.88–2.03 Å and C–N distances of 1.63–1.90 Å. The four differing calculations all give longer S–C distances (2.21–2.85 Å), but either shorter C–N distances (1.46–1.55 Å) or, in one case, a longer C–N distance of 1.97 Å.

There is only a weak correlation between the TS geometry and Δ*E* ^‡^: The correlation coefficient between the C–N distance and Δ*E* ^‡^ is 0.45 and the calculations with the longest C–N distances give the highest Δ*E* ^‡^. The correlation to the S–C distance is only 0.1 and the four outliers give Δ*E* ^‡^s of 44–146 kJ/mol.

The correlations between Δ*E* and S–C and especially the C–N distance are higher, 0.42 and –0.77. Thus, the reaction energy increases the more the fluoro-uracil group is removed from the Cys-145 residue (Figure 6). This indicates that Δ*E* has little relevance and that the studied PS is only a local minimum on the dissociation of the fluoro-uracil group or possibly its protonation by His-41. The KAC setup, with the more restricted solvation, allows the fluoro-uracil to move further away from Cys-145 than in our setup (Run1–3) and therefore gives more negative reaction energies. Likewise, relaxed surroundings allow more movements of the fluoro-uracil group than fixed surroundings, explaining why ComQum gives more negative Δ*E* than ORCA (with a smaller relaxed region).

**Figure 6:**
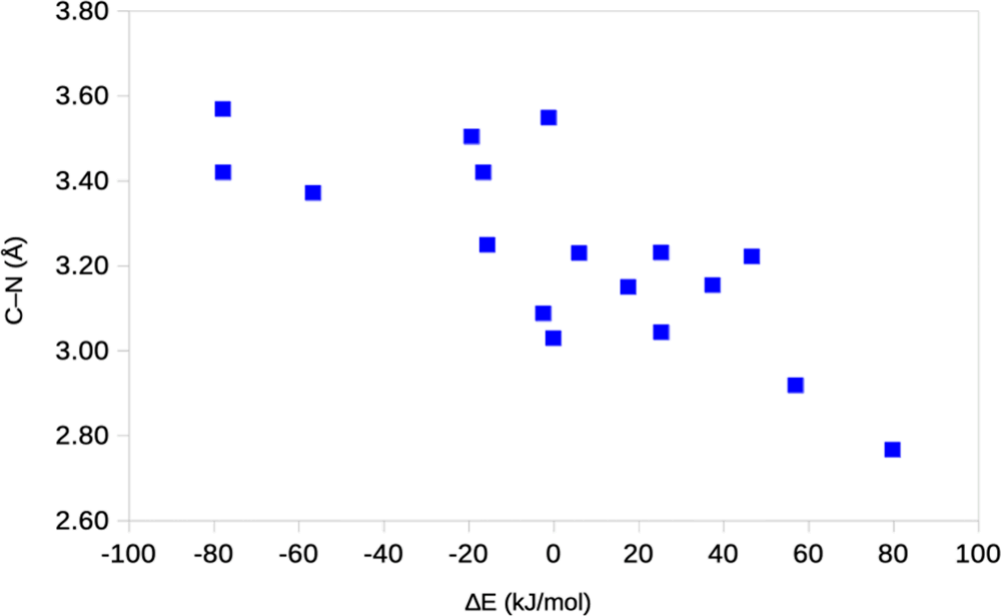
Correlation between Δ*E* and the C–N distance in the PS for the various QM/MM optimizations (raw data in Table 3).

Finally, we recalculated Δ*E* ^‡^ and Δ*E* with the big-QM approach. The results are shown in Table 4. It can be seen that the convergence with the size of the QM system is better than for the single-point calculations. The average for the range of the results obtained with *r* = 6, 8, and 10 Å is 7 kJ/mol with neutralized systems and the COSMO continuum solvation model (ε = 80/neu columns). This is better than the standard big-QM calculation with point charges (EE columns) or with the continuum solvent (ε = 80 columns), 18 and 9 kJ/mol respectively. The variation is larger for the calculations with relaxed surroundings, especially for Δ*E* ^‡^ and with ComQum, which is quite natural – with relaxed surroundings many more atoms have moved and have different coordinates in the various big-QM calculations. The number of relaxed atoms is also larger for ComQum than with ORCA. The difference between *r* = 8 and 10 Å is even smaller, 0–9 kJ/mol and only 2 kJ/mol on average for the neutralized big-QM calculations. This gives some confidence that the results at *r* = 8 and 10 Å are reasonably converged.

**Table 4:** QM/MM and Big-QM (Treating the Surroundings with an EE-QM/MM Model (eq 4, EE) or with a COSMO Continuum Solvation Model with a Dielectric Constant of 80 (ε = 80), Possibly Neutralizing Solvent Accessible Charges (neu), Using Three Different Radii, *r* = 6, 8, or 10 Å) Δ*E* ^‡^ and Δ*E* Energies (kJ/mol) for the Various QM/MM Geometry Optimizations (with Four Different Setups, Either Fixed or Relaxed MM Surroundings (Relax) and Using Two Different Software)^a^

				EE			ε = 80			ε = 80/neu		
setup	relax	software	QM/MM	*r =* 6	8	10	6	8	10	6	8	10
Δ*E* ^‡^												
KAC	yes	ComQum	44	44	41	38	34	40	43	34	55	60
		ORCA	61				123			123	117	118
	no	ComQum	70	133	127	112	122	118	119	122	117	118
		ORCA	87				121			121	115	116
Run1	yes	ComQum	108	140	151	143	162	152	144	162	158	149
		ORCA	113				104			104	94	93
	no	ComQum	92	106	113	107	101	103	103	101	100	101
		ORCA	101				92			92	87	87
Run2	yes	ComQum	132	188	168	164	137	168	164	137	154	154
		ORCA	146				144			144	141	141
	no	ComQum	108	116	120	111	113	113	112	113	110	108
		ORCA	115				101			101	93	91
Run3	yes	ComQum	123	160	164	154	138	152	154	138	152	153
		ORCA	89				65			65	63	62
	no	ComQum	73	59	64	61	62	61	60	62	57	55
		ORCA	77				64			64	58	56
Δ*E*												
KAC	yes	ComQum	–78	–50	–71	–99	–79	–84	–86	–79	–66	–63
		ORCA	–57				–21			–21	–26	–22
	no	ComQum	–16	66	49	18	40	30	29	40	32	34
		ORCA	6				40			40	31	33
Run1	yes	ComQum	–1	34	51	37	51	41	39	51	53	53
		ORCA	57				40			40	35	38
	no	ComQum	–2	40	41	26	25	26	27	25	26	28
		ORCA	80				69			69	63	64
Run2	yes	ComQum	–19	37	34	16	6	29	19	6	12	7
		ORCA	25				23			23	22	23
	no	ComQum	18	41	48	25	30	30	29	30	27	27
		ORCA	37				30			30	25	25
Run3	yes	ComQum	–17	3	7	–2	4	1	0	4	8	8
		ORCA	25				–20			–20	–20	–20
	no	ComQum	0	19	20	5	2	5	5	2	3	4
		ORCA	47				16			16	12	11

a The Z1 charge redistribution scheme was used for the EE calculations.

Concentrating on the results with neutralized charges and *r* = 10, it can be seen that big-QM does not diminish the difference between the various calculations compared to the QM/MM results. On the contrary, the variation is actually somewhat larger, 60–154 kJ/mol for Δ*E* ^‡^ and –63 to 64 kJ/mol for Δ*E*. Thus, the differences are caused by differences in the obtained geometries, rather than by the instability of the calculated energies. However, the difference between the ComQum and ORCA results with fixed surroundings decreases, especially for Δ*E* (from 43 to 11 kJ/mol on average), showing that it is mainly caused by the difference in the charge-redistribution scheme.

## Conclusions

We have studied the reproducibility of QM/MM calculations using the formation of a covalent adduct of carmofur with SARS-CoV-2 main protease as a test case. Krylov and co-workers (KAC) have studied this system before and reported differences of up to 30 kJ/mol between QM/MM implementations in NWChem and Q-Chem for single-point energy calculations on the same structure.^9^ Here, we show that a similar range is observed also for QM/MM implementations in ComQum, ORCA and AMBER. The main reason for this difference is the charge-redistribution scheme, i.e. which point charges are included around the HL atoms in the QM calculations. The default approach in ComQum is Z1, i.e. all charges are kept except that of the CL atom (which is the same as the HL atom in the QM calculations), whereas it is the charge-shift (CS) scheme in ORCA, where charges (including that on CL) are moved away from HL, keeping the charge and dipole. If the same charge-redistribution scheme is employed, calculations with ComQum and Amber differ by only 6–10 kJ/mol. The remaining differences probably come from the fact that ComQum employs a subtractive scheme, whereas ORCA employs an additive scheme. However, QM/MM calculations with an additive scheme in ComQum gives the same results as the additive scheme within 0.1 kJ/mol and variants of the additive scheme in ORCA also only change Δ*E* by only ± 1 kJ/mol, indicating some additional differences in the QM/MM implementation in the two software.

Thus, it is important to specify details of the QM/MM calculations: the scheme (additive or subtractive), the embedding (mechanical, electrostatic or polarized), the charge-redistribution scheme and HL atom corrections. Moreover, it is important for each software to specify exactly how QM/MM was implemented. In particular, it is important to specify exactly which terms are included in calculations with the additive scheme, details of the charge-redistribution scheme (in particular CS is poorly described in the original publication^8^) and exactly how the HL and CL atoms are treated.

Both the large effect of the charge-redistribution scheme and the large size of the MM term indicates that the 83-atom QM region employed is too small. Therefore, we employed the big-QM approach to obtain energies that are converged with respect to the size of the QM region. We suggest that our original big-QM scheme should be enhanced with the additional rule that solvent-exposed charged residues should be neutralized in the big-QM region. With this addition, the big-QM energies seem to be reasonably converged at a radius of 8 Å (∼1400 atoms). For this solvent-exposed active site, the convergence of the big-QM calculations is poorer than in the previous test cases^16,30,31^ and calculations in a continuum solvent give the most stable results.

Third, we have investigated how different setups of the protein for QM/MM affect the results. KAC used mainly a single shell of water molecules around the protein (cf. Figure 1a) and relaxed the protein, whereas we use a sphere of water molecules, at least 10 Å from any protein atom (Figure 1b) and keep the heavy atoms of the protein fixed at the crystal positions. The two setups give widely different results, differing by up to 88 kJ/mol for Δ*E* ^‡^ and by 114 kJ/mol for Δ*E*. This reflects that the KAC setup, with its restricted solvation, allows for larger relaxation of the structure than our setup.

For our spherical setup, we solvated the protein with three different water boxes, providing three equally likely setups of the protein. For each we performed three independent optimizations of the three steps in the reaction (RS, TS and PS). This gave variations of up to 57 kJ/mol in Δ*E* ^‡^ and 42 kJ/mol in Δ*E*. Moreover, there are large differences between results obtained with relaxed or fixed surroundings. Both observations reflect that the reaction takes place at the surface of the protein and therefore is very sensitive to the solvent structure. It is an unavoidable problem for methods involving explicit solvation and minimized structures and indicates that such a reaction is better studied by free-energy methods like umbrella sampling or metadynamics.

## Supplementary Material



## Notes

Description of the treatment of hydrogen link atoms in ComQum; example figures of the sizes of the big-QM systems; AMBER topology file for carmofur; sample ORCA input file for QM/MM with relaxed surroundings; sample AMBER input file for a single-point QM/MM calculation; number of relaxed atoms in the QM/MM calculations. Coordinates for the QM/MM optimized structures have been uploaded to Zenodo, DOI: 10.5281/zenodo.15812391 (PDF)
