# Supplementary material for: Reproducibility of QM/MM Calculations for the SARS-CoV‑2 Main Protease
Source: J Chem Theory Comput. 2025 Jul 24;21(15):7711–23. doi: 10.1021/acs.jctc.5c00841 (PMC12355682; doi:10.1021/acs.jctc.5c00841)
Supplement: Supplementary file 1 [file ct5c00841_si_001.pdf]

Supporting Information

On the reproducibility of QM/MM calculations for SARS-CoV-2 main protease

Xiaoli Sun [a,b] and Ulf Ryde [*,a]

[a] Department of Computational Chemistry, Lund University, Chemical Centre, P. O. Box 124, SE-221 00 Lund, Sweden

[b] Institute of Theoretical Chemistry, College of Chemistry, Jilin University, Changchun, 130012, PR China

Correspondence to Ulf Ryde, E-mail: Ulf.Ryde@compchem.lu.se,

Tel: +46 – 46 2224502

2025-07-05

Description of the treatment of HL atoms in ComQum

QM calculations require filled valences. Therefore, the QM region needs to be truncated in a proper way. ComQum uses the hydrogen link-atom approach,[1, 2] in which the QM region is truncated by adding a hydrogen atom for each QM–MM bond, called the hydrogen link atom (HL), which typically replaces a carbon atom in the MM region, called the carbon link atom (CL). The positions of the HL atoms are unambiguously determined by the positions of the corresponding CL atoms according to

$$\bar{r}_{\mathrm{HL}} = \bar{r}_{\mathrm{Q1}} + g_{\mathrm{b}}(\bar{r}_{\mathrm{CL}} - \bar{r}_{\mathrm{X}}) \tag{S2}$$

where Q1 is the atom in the QM system to which HL or CL is bound (cf. Figure 3 in the main article) and g_{b} is a precalculated parameter for each kind of bond, obtained as the quotient between the ideal Q1–HL bond distance calculated with the QM method employed for a model system of the truncated residue and the ideal Q1–CL bond distance according to the MM force field.[1]

The 83-atom QM region employed in this study contains four HL atoms: CA–CB of His-41, N–CA and CA–CB of Asn-142, as well as CA–C of Cys-145. For these four atoms, we used g_{b} factors of 0.7215, 0.7598, 0.7215 and 0.7234.

Figure S1. The three sizes of the big-QM systems: a) r = 6 Å, b) r = 8 Å and c) r = 10 Å. The original 83-atom QM region is shown in a ball-and-stick model.

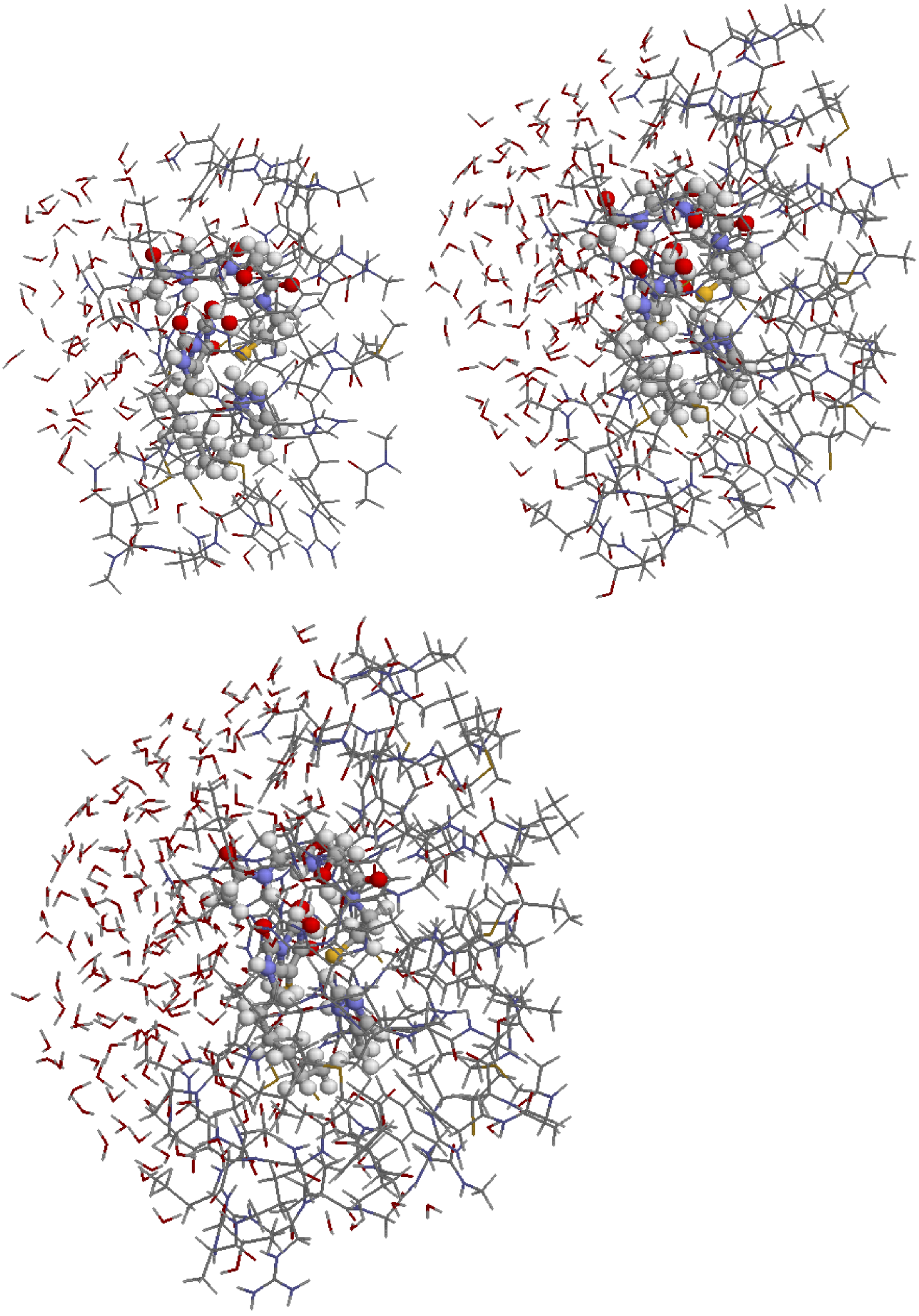

Table S1. AMBER topology (jry.in) file for carmofur. The third column contains the GAFF2 atom types and the last column the AM1-BCC charges.

```
    0    0    2

Carmofur, AM1-BCC charges.
molecule.res
JRY   INT  0
CORRECT     OMIT DU   BEG
  0.0000
   1  DUMM  DU    M    0  -1  -2     0.000      .0        .0      .00000
   2  DUMM  DU    M    1   0  -1     1.449      .0        .0      .00000
   3  DUMM  DU    M    2   1   0     1.523   111.21       .0      .00000
   4  O4    o     M    3   2   1     1.540   111.208  -180.000 -0.616500
   5  C4    c     M    4   3   2     1.212   100.983   -83.681  0.814500
   6  N3    n     M    5   4   3     1.385   120.668   -96.994 -0.583500
   7  H3    hn    E    6   5   4     1.029   114.286   -12.109  0.360500
   8  C2    c     M    6   5   4     1.378   129.292   171.200  0.702700
   9  O2    o     E    8   6   5     1.226   122.694  -174.185 -0.570500
  10  C1    cc    M    8   6   5     1.444   111.590     6.540 -0.018700
  11  F1    f     E   10   8   6     1.344   117.014   179.866 -0.128500
  12  C6    cd    M   10   8   6     1.344   121.936     2.865  0.009600
  13  H6    h4    E   12  10   8     1.080   120.155   170.568  0.198000
  14  N5    n     M   12  10   8     1.375   122.152    -8.803 -0.415600
  15  C7    c     M   14  12  10     1.450   121.106   179.850  0.791100
  16  O7    o     E   15  14  12     1.224   118.704  -148.087 -0.591100
  17  N7    n     M   15  14  12     1.330   118.442    23.787 -0.521900
  18  H7    hn    E   17  15  14     1.021   110.398  -166.904  0.335500
  19  C8    c3    M   17  15  14     1.453   131.982    28.707  0.084000
  20  H82   h1    E   19  17  15     1.093   108.889     6.066  0.072200
  21  H83   h1    E   19  17  15     1.094   107.680   118.529  0.072200
  22  C9    c3    M   19  17  15     1.530   111.577  -118.237 -0.110400
  23  H92   hc    E   22  19  17     1.095   109.299   -77.431  0.046200
  24  H93   hc    E   22  19  17     1.097   109.916    37.044  0.046200
  25  C0    c3    M   22  19  17     1.520   111.963   160.087 -0.078400
  26  H02   hc    E   25  22  19     1.097   109.603  -173.592  0.047200
  27  H03   hc    E   25  22  19     1.102   108.448   -58.027  0.047200
  28  C11   c3    M   25  22  19     1.523   112.491    63.951 -0.079400
  29  H111  hc    E   28  25  22     1.097   109.471   -52.903  0.039200
  30  H112  hc    E   28  25  22     1.098   108.591    62.866  0.039200
  31  C12   c3    M   28  25  22     1.523   113.476  -174.152 -0.079400
  32  H121  hc    E   31  28  25     1.094   109.367  -166.056  0.040700
  33  H122  hc    E   31  28  25     1.098   110.327   -49.160  0.040700
  34  C13   c3    M   31  28  25     1.518   111.429    73.975 -0.094100
  35  H131  hc    E   34  31  28     1.091   112.085  -172.813  0.033700
  36  H132  hc    E   34  31  28     1.094   109.489    66.556  0.033700
  37  H133  hc    E   34  31  28     1.097   111.430   -52.368  0.033700


LOOP
   N5   C4

IMPROPER
   N5   N3   C4   O4
   C4   C2   N3   H3
   C1   N3   C2   O2
   C2   C6   C1   F1
   C1   H6   C6   N5
   C7   C4   N5   C6
   N5   N7   C7   O7

DONE
STOP
```

Table S2. Number of relaxed atoms in the various calculations.

	ComQum	ORCA
KAC	891	616
Run1	741	534
Run2	784	559
Run3	762	561

Table S3. Sample ORCA input file for QM/MM with relaxed surroundings.

```
!QMMM PBE0 6-31G* D3ZERO Opt
%PAL NPROCS 48 END

%qmmm
    ORCAFFFilename   "prmtop3.ORCAFF.prms"
    QMAtoms          { 608:619
                       2204:2205
                       2214:2241
                       4682:4715
                       4716:4718 } end
    ActiveAtoms      { 608:619
                       2204:2205
                       2214:2241
                       4682:4715
                       4716:4718
                       354 355 357 375 380 381 382 383 389 390
                       391 392 404 405 406 570 571 575 602 604
                       605 606 607 620 621 622 625 662 727 728
                       729 730 731 732 733 734 811 812 1840 1844
                       1853 1854 1856 1857 1858 1859 2167 2169 2171 2172
                       2173 2183 2184 2185 2200 2201 2202 2203 2206 2207
                       2208 2209 2210 2242 2243 2244 2245 2246 2251 2252
                       2253 2255 2256 2258 2259 2496 2502 2503 2504 2505
                       2506 2509 2510 2513 2515 2522 2523 2526 2527 2528
                       2529 2530 2534 2841 2842 2844 2849 2850 2851 4719
                       4720 4721 4722 4724 4725 4726 4727 4728 4729 4730
                       4770 4771 4772 4911 4912 4913 5067 5068 5069 5109
                       5110 5111 5205 5207 6309 6311 6822 6823 6824 9313
                       9684 9686 13639 18060 18061 18062 20895 20896
23300 } end
      ExtendActiveRegion        distance
      Dist_AtomsAroundOpt       2.
      PrintOptRegionExt         true
end

*pdbfile 0 1 pdb3.pdb
```

Table S4. Sample AMBER input file for a single-point QM/MM calculation.

```
QM/MM sp calculation on 83REAC_density_espfit.pdb, 8/11-23
 &cntrl
  irest=0,ntx=1,
  nstlim=1,dt=0.0,
  temp0=0.0,ntt=1,tautp=0.2,
  ntc=1,ntf=1,
  nsnb=25,cut=1000.0,dielc=1.0,
  ntpr=1,ntwx=0,ntwv=0,ntwe=1,ntxo=1,
  ntb=0,ntp=0,taup=0.2,
  ifqnt=1
 &end

 &qmmm
  qmmask='@0-33,642-653,2238-2239,2248-2275,4716-4718'
  qmcharge=0,spin=1
  qm_theory="EXTERN",writepdb=1
 &end

 &orc
  method = 'pbe0',
  basis='6-31g*',
  num_threads=24,
 convkey='D3ZERO',
 maxiter=5000,
 &end
```

References

(1) Ryde, U. The coordination of the catalytic zinc in alcohol dehydrogenase studied by combined quantum-chemical and molecular mechanics calculations. *J. Comput-Aided Mol. Design* **1996**, *10*, 153-164.

(2) Reuter, N.; Dejaegere, A.; Maigret, B.; Karplus, M. Frontier Bonds in QM/MM Methods: A Comparison of Different Approaches. *J. Phys. Chem. A* **2000**, *104*, 1720-1735. DOI: 10.1021/jp9924124.
